# Elevated IgA and IL-10 levels in very-early-onset inflammatory bowel disease secondary to IL-10 receptor deficiency

**DOI:** 10.1590/1984-0462/2022/40/2020434

**Published:** 2021-10-29

**Authors:** Natascha Silva Sandy, Lia Furlaneto Marega, Giane Dantas Bechara, Adriana Gut Lopes Riccetto, Carmen Bonfim, Maria Marluce dos Santos Vilela, Antonio Fernando Ribeiro, Maria De Fatima Servidoni, Elizete Aparecida Lomazi

**Affiliations:** a University of Toronto, Toronto, ON, Canada.; b Universidade de Campinas, Campinas, SP, Brazil.; c Hospital Pequeno Príncipe, Curitiba, PR, Brazil.

**Keywords:** Inflammatory bowel diseases, Whole exome sequencing, Genetic techniques, Primary immunodeficiency diseases, Child, Doenças inflamatórias intestinais, Sequenciamento completo do exoma, Técnicas genéticas, Doenças da imunodeficiência primária, Criança

## Abstract

**Objective:**

To report two patients with very-early-onset inflammatory bowel disease (VEOIBD) secondary to interleukin-10 receptor (*IL-10R*) mutations, explore immunophenotyping data and plasma cytokine profile on these cases compared to healthy controls, and describe the phenotype of *IL-10*/*IL-10R* mutations based on a literature review.

**Case description:**

We report on two female infants referred to our tertiary center at the age of ten months, with severe colonic and perianal disease, as well as significant malnutrition, who had shown limited response to usual inflammatory bowel disease (IBD) therapy agents. In the first case, whole-exome sequencing (WES) revealed a homozygous (c.537G>A/p.T179T) mutation in exon 4 of the *IL-10RA* gene, while in the second patient, compound heterozygosity was identified, also in the *IL-10RA* gene (chr11:117.859.199 variant A>G/p.Tyr57Cys and chr11: 117.860.335 variant G>T/p.Val123Leu). Both patients underwent hematopoietic cell transplantation (HCT). Immunological work-up of these patients revealed increased IL-10 plasma levels and increased IgA.

**Comments:**

Our case reports disclose novel findings on plasma cytokine profile in *IL-10R* deficiency, and we describe the severe phenotype of *IL-10*/*IL-10R* deficiency that should be recognized by physicians.

## RESUMO

**Objetivo:**

Relatar os casos de duas pacientes com doença inflamatória intestinal de início muito precoce (em inglês VEOIBD) secundária a mutações do receptor de interleucina 10 (*IL-10R*), explorar dados de imunofenotipagem e perfil de citocinas plasmáticas nesses casos em comparação com indivíduos saudáveis e descrever o fenótipo de mutações *IL-10/IL-10R* com base em uma revisão da literatura.

**Descrição do caso:**

Duas lactentes do sexo feminino foram encaminhadas ao nosso centro terciário, ambas com dez meses no momento do encaminhamento, com doença colônica e perianal grave, bem como desnutrição significativa, tendo uma resposta limitada aos agentes de terapia usuais de doença inflamatória intestinal (DII). No primeiro caso, o sequenciamento completo do exoma revelou mutação homozigótica (c. 537G>A/p.T179T) no exon 4 do gene *IL-10RA*, enquanto no segundo caso heterozigosidade composta foi identificada também no gene *IL-10RA* [chr11: 117.859.199 - variante A>G/p.Tyr57Cys e chr11: 117.860.335 - variante G>T/ p.Val123Leu]. Ambas as pacientes foram submetidas a Transplante de Células-Tronco Hematopoiéticas. A investigação imunológica das pacientes revelou aumento dos níveis plasmáticos de IL-10 e aumento da IgA.

**Comentários:**

Nossos relatos de casos descrevem novos achados no perfil de citocinas plasmáticas na deficiência de IL-10R, e relatamos o fenótipo grave da deficiência de *IL-10/IL-10R* que deve ser reconhecido pelos médicos.

## INTRODUCTION

Very-early-onset inflammatory bowel disease (VEOIBD) is defined as inflammatory bowel disease (IBD) presenting at an age younger than six years.^1^ The importance of this classification relates to the fact that pediatric patients with IBD have many age-dependent characteristics, such as: likelihood of an underlying monogenic etiology, disease anatomic location, severity, and response to therapy.^1^ ^,^ ^2^ ^,^ ^3^ Although VEOIBD represents a relatively small fraction of pediatric IBD - around 15%,^4^ it is reported to be the most rapidly growing subgroup in incidence.^5^ VEOIBD commonly presents with pancolitis, malnutrition/growth failure, and severe and refractory disease,^6^ which can be associated with known family history/genetic background or may be a *de novo* monogenic disease. A variety of mechanisms have been identified in VEOIBD, including but not limited to: epithelial barrier dysfunction, defects in phagocytes, B and T cells, immune dysregulation, signaling defects related to IL-10, and hyperinflammatory disorders.^7^ The dysregulation of the intestinal immune system is an important element in the pathogenesis of IBD:^8^ the imbalance between pro- and anti-inflammatory cytokines is one of the key pathophysiological elements for inflammation initiation, progression, and resolution.^9^ ^,^ ^10^ The roles of cytokines in IBD as potential markers and/or therapeutic targets may provide an effective approach to the long-term control of this inflammation.^9^ ^,^ ^10^

A growing number of genes have been implicated in monogenic forms of VEOIBD,^11^ and next-generation sequencing techniques have truly revolutionized its diagnosis. Unfortunately, the limited availability of this technology associated with the complexity of the VEOIBD course often results in a significant delay in initiation of specific/directed therapy, leading to increased morbidity. More than 200 susceptibility loci have been connected with the pathogenesis of polygenic IBD,^12^ with more than 50 involved in monogenic IBD-like phenotypes,^1^ including interleukin-10 (*IL-10*), IL-10 receptor (*IL-10R*), X-linked inhibitor of apoptosis protein (*XIAP*), and *FOXP3* genes.^7^ ^,^ ^13^

We aim to report the first two patients with VEOIBD and *IL-10R* mutations diagnosed at our institution, explore immunophenotyping data and plasma cytokine profile on these cases, and describe the phenotype of *IL-10*/*IL-10R* mutations based on a literature review.

## CASE REPORT

We report on two patients diagnosed with *IL-10R* mutations followed at the Hospital de Clínicas da Universidade Estadual de Campinas - Department of Pediatrics, who underwent hematopoietic cell transplantation (HCT) under the Bone Marrow Transplantation Service at the Hospital de Clínicas da Universidade Federal do Paraná (UFPR). Data were acquired based on a retrospective review of their medical records, as well as prospective clinical and laboratory documentation, including demographics, clinical data, and endoscopic and laboratory findings. In both cases, legal guardians (parents) agreed to participate and signed an informed consent form. All methods were carried out in accordance with our institution’s Research Ethics Committee (REC) guidelines and regulations: the study was endorsed by our REC under the approval number 3,655,828.

For the cytokine profile analysis, multiplex assays were conducted based on 4 mm of ethylenediaminetetraacetic acid (EDTA) anticoagulated blood samples collected from 5 healthy controls - as laboratories in each center have to establish their own references values - and from both *IL-10RA* patients for plasma collection by centrifugation (1500×g, 10 min). Plasma levels of IL-2, IL-5, IL-6, IL-8 (CXCL8), and IL-10 were analyzed using the MILLIPLEX MAP Human High Sensitivity T Cell Panel - Immunology Multiplex Assay^®^ (Merck - Darmstadt, Germany), according to the manufacturer’s instructions. The samples were run in duplicate using the Bio-Plex Manager software, version 6.0 (Bio-Rad^®^). The 5 controls were healthy patients, aged 10-15 years - selected as a convenience sample, since the levels of these studied cytokines do not differ between children and adolescents.^14^

### CASE 1

A ten-month-old female was first referred to pediatric surgery with the diagnosis of anorectal malformation. Past medical history was relevant for a colostomy at the age of two months - cow’s milk protein allergy (CMPA) and recurrent infections, including multiple sepsis episodes secondary to urinary tract infections, skin infections, and Fournier gangrene. On assessment, she was pale, tachycardic, and severely malnourished; anorectal examination revealed anal stenosis and an active perianal fistula. Laboratory data at referral showed severe anemia (hemoglobin of 6.2 g/dL), persistently elevated inflammatory markers, and thrombocytosis. Fecal calprotectin was 3,300 µg/g.

She was admitted for supportive care, further investigation, and nutritional rehabilitation. Colonoscopy demonstrated chronic pancolitis, compatible with IBD. Treatment was started with steroids, followed by azathioprine and infliximab, with limited response. Whole-exome sequencing (WES) revealed a homozygous (c.537G>A, p.T179T) mutation in exon 4 of the *IL-10RA* gene. The same mutation was found in heterozygosity in both parents, characterizing autosomal recessive inheritance. She was referred for HCT at the age of 6 years, although still severely malnourished (weight 12.8 kg) and with a chronically active infection at the skin surrounding the colostomy.

She underwent a matched unrelated (12/12) bone marrow transplantation from an ABO compatible, CMV negative, 25-year-old male donor. Myeloablative preparative regimen included busulfan 18 mg/kg, fludarabine 160 mg/m^2^, and rabbit ATG (thymoglobuline^®^) 6 mg/kg. Graft versus host disease (GVHD) prophylaxis with cyclosporine and mycophenolate mofetil (MMF) was used. The total nucleated and CD34 cell doses infused were 5.7×10^8^/kg and 3.9×10^6^/kg, respectively. On day +7 post-HCT, she presented with upper respiratory infection symptoms, and a nasopharyngeal swab tested positive for parainfluenza (negative for COVID-19). Mucositis grade II was detected on day +10. Neutrophil engraftment was noted on day +14, and platelet engraftment on day +26. Chimerism analysis initially revealed a mixed chimerism (day +35 - total donor: 79%; CD3: 33%; CD33: 90%), immunosuppression was maintained with adequate levels of cyclosporine, and repeated analysis showed progressive donor chimerism (day +90 - total donor: 88%; CD3: 61%; CD19: 100%; CD33: 96%). She is currently in excellent clinical condition, with no complications related to the underlying disease or the HCT.

On the immunological work-up of this patient, we observed elevated CD8^+^ peripheral blood lymphocytes, with an inversion ratio of CD4^+^/CD8^+^ and reduced CD19^+^ and CD16^+^CD56^+^. Additionally, we identified high IL-8, IL-6, and IL-10 plasma levels, decreased IL-2 plasma levels, and increased IgA levels.

### CASE 2

A ten-month-old female was referred to the pediatric gastroenterology clinic with a history of non-bloody diarrhea and failure to thrive starting at three months of age. She had severe anemia (hemoglobin: 7.3 g/dL) and chronic malnutrition (albumin 1.7 g/dL). Fecal calprotectin was 5,288 µg/g. Lower endoscopy demonstrated severe active colitis; however, examination was limited to the sigmoid, where a stenotic segment was present and could not be transposed. She had been started on steroids with minimal symptomatic improvement. WES identified 2 heterozygous mutations in the *IL-10RA* gene (chr11: 117,859,199 - variant A>G, resulting in p.Tyr57Cys and chr11: 117,860,335 - variant G>T, resulting in p.Val123Leu). The first variant was identified in the patient’s mother, while the second was not found in the parents, and therefore, was characterized as *de novo*. She was referred for HCT assessment.

Prior to HCT, she received 10 days of systemic antibiotics due to an infection related to a rectovaginal fistula and 14 days of low-molecular-weight heparin for occlusive central venous catheter-related thrombosis. She underwent HCT at the age of 23 months, also severely malnourished (weight 6.5 kg). The same myeloablative preparative regimen described in case 1 was used. The stem cell source was an ABO compatible, CMV positive, unrelated cord blood (9/10, *locus* B mismatch), and the total nucleated and CD34 cell doses infused were 17×10^7^/kg and 3.5×10^5^/kg, respectively. The post-HCT course was complicated by mucositis grade II (seen on day +13) and BK-negative hemorrhagic cystitis (on day +20). Neutrophil engraftment was noted on day +18, and platelet engraftment on day +40. Donor chimerism on day +30 showed 100% of donor cells. Due to an ongoing active perianal disease and given the risk of infection, the patient underwent a diverting colostomy approximately a month after HCT, which allowed healing of her perianal disease. She has experienced gradual improvement of her underlying colitis, and her chronic diarrhea has resolved. She has not experienced other complications related to the underlying disease or complications related to the HCT.

Immunophenotyping of this patient showed reduced CD8^+^ peripheral blood lymphocytes, while plasma cytokine profile analysis revealed elevated IL-10 plasma levels. Also, IgA levels were elevated, and neutrophilia was observed.

A summary of genetic information and immunological characterization of the two cases reported herein, as well as a comparison with data from a case previously described in the literature,^15^ is provided in Table 1.

**Table 1:** Genetic information and laboratory characteristics.

	Case 1		Reference values for 2-6-year-olds	Case 2	Reference values for 1-2-year-olds	Case 3^15^	Reference values
Gender	F			F		M	
Surgical therapy	Colostomy			Colostomy		Ileostomy	
Mutation	Thr179Thr			Tyr57Cys and Val123Leu		Tyr57Cys	
Mother	Thr179Thr			Tyr57Cys			
Father	Thr179Thr						
Age at examination	3 y	5 y		1 y			
Platelets (×10^3^)	628	358	150-400	747	150-450		
WBC/μL (×10^3^)	10.4	10.32	4-10	21.9	4-10		
Neutrophils/μL	5730	6250	2000-8000	17820	2000-8000		
Monocytes/μL	832	320	200-800	610	200-800		
Lymphocytes/μL	3796	3690	2210-5804	3360	3245-6981		
CD19^+^/mm^3^	657	49	328.2-1079.5	706	648-2072.3	610	200-2100
CD19+ (%)	14.5	1.4	13.3-26.7	30.3	15.6-32.2	18	14-44
CD3^+^/mm^3^	3348	3431	1498.4-3815.7	1079	1906.9-4313.9	2390	900-4500
CD3^+^ (%)	73.9	97.2	57.1-71.7	46.3	52.6-69.4	70	43-76
CD3^+^CD4^+^/mm^3^	2173	1163	786.2-2085.5	665	957.2-2727.1	1430	500-2400
CD3^+^CD4^+^ (%)	64.9	33.9	27.7-46.3	61.6	26.1-47.0	42	23-48
CD3^+^CD8^+^/mm^3^	803	2059	452.3-1700.5	350	563.3-1753.2	890	300-1800
CD3^+^CD8^+^ (%)	24	60	15.7-33.8	32.4	14.4-27.5	26	14-33
CD16^+^CD56^+^/mm^3^	435	25	134.6-600.8	536	153.0-702.9	340	100-1000
CD16^+^CD56^+^ (%)	9.6	0.7	7.8-16.1	23	3.3-14.6	10	4-23
CD3^+^CD16^+^CD56^+^/mm^3^	157	130		32			
CD3^+^CD16^+^CD56^+^ (%)	4.7	3.8		3			
CD4/CD8 ratio	2.70	0.56	>1.5	1.9	>1.5	1.6	0.9-2.9
TCRab^+^CD3^+^CD4^-^CD8^-^ (%)	1	n/a	<1.5	n/a	<1.5		
IgM (mg/dL)	141	202	24-276	149	28-173	130	50-220
IgA (mg/dL)	731	815	33-308	178	24-184	250	30-120
IgG (mg/dL)	1770	1500	630-2000	507	410-1630	874	310-1380
IgE (IU/mL)	30.2	n/a		n/a			
IL-10 (pg/mL) ^#^		54.58		51.2	25.2 (22.6-41.2)		
IL-6 (pg/mL) ^#^		19.3		3.31	2.4 (0.7-8.4)		
IL-8 (pg/mL) ^#^		31.17		5.83	5.1 (3-7.1)		
IL-2 (pg/mL) ^#^		*0.28		1.34	0.7 (0.6-1.6)		
IL-5 (pg/mL) ^#^		12.49		10.06	9.8 (7.0-13.5)		

WBC: white blood cells; CD: cluster of differentiation; Ig: immunoglobulin. IL: interleukin; n/a: not available; μL: microliters; mm^3^: cubic millimeters; mg/dL: milligrams per deciliter; pg/mL: picogram per deciliter; IU/mL: international units per milliliter. *value out of range (below the detection limit of the assay), hence requiring extrapolation calculation. ^#^Same reference values for both cases.

## DISCUSSION

Since Glocker et al.’s initial report in 2009,^16^ around 100 scattered reports on *IL-10* or *IL-10R* mutations related to VEOIBD have been documented; however, reports from South America are scarce.^17^ ^,^ ^18^ ^,^ ^19^ The IL-10 pathway has been well established as central to the modulation of inflammation in the gastrointestinal tract. IL-10 is a multifunctional cytokine produced by different subtypes of leukocytes (in response to numerous stimuli); it has tissue-specific effects exerted by specific tetrameric receptors, mainly determining activation of the IL-10/JAK1/STAT3 cascade.^20^ ^,^ ^21^

IL-10R has two units, alpha (IL-10RA) and beta (IL-10RB), and receptor defects are reported in both subunits: more often in the alpha subunit;^17^ nevertheless, receptor compound heterozygous mutations (*IL-10RA*/*IL-10RB*) and homozygous *IL-10RB* mutations have also been described.^17^ ^,^ ^22^ ^,^ ^23^ IL-10R deficiency, or even its functional impairment, leads to abnormal signaling determining an IL-10 deficiency phenotype.^24^ Mutations in *IL-10* and its receptor have been mainly reported in Europe, North America, and Asia. Prior to this publication, in our literature review, we could only identify one case from Brazil (*IL-10RA* mutation), reported as part of an international collaboration.^23^

As in the two cases reported herein, patients with *IL-10* or *IL-10R* mutations typically present with extremely early onset of symptoms, severe colitis and perianal disease, severe infectious diseases, and marked failure to thrive.^17^ ^,^ ^23^ ^,^ ^25^ ^,^ ^26^ Eczema, folliculitis, and oral ulcers are also often reported.^17^ Response to usual IBD therapy agents, either as single-agent or combined therapies, is typically poor.^17^ ^,^ ^26^ Use of steroids, as in our two patients, is common among this VEOIB subpopulation, with minimal symptomatic relief. Response to different medical therapies, if present at all, is often not sustained.^23^ ^,^ ^24^ ^,^ ^25^ Management of perianal disease frequently requires colectomy/ileostomy.^24^ So far, the only definitive/curative treatment reported is HCT,^16^ ^,^ ^23^ ^,^ ^25^ and, thus, this was the modality chosen for both cases in this report.

We disclose a novel finding of high levels of IL-10, present in both cases when compared to our controls and also to the reference data previously described in the literature.^14^ Additionally, we detected increased IgA levels in our cases, as previously described by Engelhardt et al.^15^ Furthermore, in our first case, we noted high levels of IL-6 and IL-8, a finding that, to be best of our knowledge, has not been previously reported in *IL-10*/*IL-10R* mutations. As we know, IL-10 is a pleiotropic and important immunoregulatory cytokine^8^ and has been associated with impaired viral clearance or viral persistence,^27^ while IL-6 and IL-8 are pro-inflammatory cytokines involved in the differentiation and proliferation of a variety of cells and in tissue damage.^8^ Data on more patients with *IL-10*/*IL-10R* deficiency, perhaps from a multicenter study, would be necessary to further understand the imbalance between pro- and anti-inflammatory cytokines, as they may be relevant biomarkers. Moreover, investigations into these critical intercellular signaling molecules in *IL-10* and *IL-10RA* variants could provide important information about cytokine dysregulation.^28^

In conclusion, our case reports disclose novel findings on plasma cytokine profile in *IL-10R* deficiency, and we describe the severe phenotype of *IL-10*/*IL-10R* deficiency that should be recognized by physicians. Among monogenic causes for VEOIBD, we sought to raise awareness of the *IL-10*/*IL-10R* deficiency phenotype, as they are the most frequently identified in association with infantile-onset IBD.^17^ ^,^ ^23^ As illustrated in the cases, even in situations with no history of consanguinity or family history, *IL-10* or *IL-10R* mutations can be inherited in an autosomal recessive pattern or result from compound heterozygosity, including *de novo* mutations. With a high suspicion index, targeted analysis aimed at a specific diagnosis is possible, allowing a significant cost reduction compared to WES.^26^ ^,^ ^29^ Further investigations into cytokine dysregulation and its pathophysiological implication for VEOIBD secondary to *IL-10*/*IL-10R* deficiency are needed.
